# Soy Isoflavone Supplementation in Sow Diet Enhances Antioxidant Status and Promotes Intestinal Health of Newborn Piglets

**DOI:** 10.3390/ani15152223

**Published:** 2025-07-28

**Authors:** Le Liu, Lizhu Niu, Mengmeng Xu, Qing Yu, Lixin Chen, Hongyu Deng, Wen Chen, Long Che

**Affiliations:** 1 College of Animal Science and Technology, Henan Agricultural University, No. 15 Longzi Lake University Campus, Zhengzhou 450046, China; 18638964588@163.com; 2 College of Animal Science and Technology, Henan University of Animal Husbandry and Economy, No. 6 North Longzihu Road, Zhengdong New District, Zhengzhou 450046, China; lizhu_niu@foxmail.com (L.N.); xumengmeng2013@126.com (M.X.); 15837174986@163.com (Q.Y.); chenlixin2002@126.com (L.C.); denghongyu2004@126.com (H.D.)

**Keywords:** soy isoflavones, sow, newborn piglet, placenta, intestinal health

## Abstract

This study aimed to explore the effects of dietary supplementation with soy isoflavones (SI) in the later stages of pregnancy on the antioxidant capacity of sows and intestinal health of newborn piglets. Forty sows with similar body weights and parity (average of 1–2 parity) were randomly divided into two groups (*n* = 20): the control group and SI group (dose: 100 mg/kg of feed). Feeding was started on day 85 of gestation and continued until farrowing. SI supplementation significantly increased the antioxidant levels in the serum of the sows and newborn piglets, placental tissue, and the intestinal tract of the piglets. This observation was indicated by a decreased activity of the oxidative stress marker malondialdehyde (MDA); increased activity of antioxidant enzymes such as superoxide dismutase, glutathione peroxidase, and catalase; and enhanced total antioxidant capacity. The organ indices of the intestine and liver and the villus height/crypt depth of the jejunum of newborn piglets significantly increased. SI supplementation activated the Nrf2 signaling pathway in the jejunum of neonatal piglets and the expression of placental antioxidant proteins, and it downregulated the expression of the Bax and Caspase 3 apoptotic proteins in the placenta and neonatal piglets. Intestinal and placental barrier integrity was strengthened. For example, ZO-1, Occludin, and Claudin 1 exhibited elevated expression. In conclusion, dietary supplementation with SI enhanced the antioxidant capacity of sows and piglets and improved the health of the placenta and intestinal tract of newborn piglets.

## Simple Summary

This study intended to assess whether supplementing the diet of sows with soy isoflavones (SIs) during late gestation could improve the antioxidant capacity and health status of the offspring. The results showed improvement in both the antioxidant capacity and health status of the placenta and intestines of the newborn piglets. This study confirms that SI can influence the intestinal health of the offspring via the maternal route and explains possible regulatory mechanisms.

## 1. Introduction

In the context of modern pig farming, intestinal health in piglets serves as a fundamental basis for their growth and development, as well as economic efficiency. Intestinal development begins in the early embryonic stage. Endodermal cells initially differentiate to form the rudiment of the primitive gut tube [1]. Undifferentiated cells first form columnar epithelial villi and then differentiate into intestinal epithelial cells. Proliferating cells gradually form primitive crypts [2,3]. During this period, the healthy development of the piglet’s intestine is conducive to the absorption of nutrients. It further helps form a strong intestinal barrier, effectively resisting viral and microbial invasion. Moreover, the intestines are a key area of immune regulation, as they constantly interact with external substances to build an early intestinal immune system [4,5]. However, newborn piglets frequently encounter problems, such as poor intestinal development and intestinal dysfunction [6].

An in-depth exploration revealed that this phenomenon might be due to oxidative stress in the later stages of pregnancy in sows. During pregnancy, the mother precisely coordinates changes in the anatomical structure, physiological functions, and metabolic status to meet the nutritional and oxygen requirements of fetal development [7]. Additionally, the direct physical exchange and metabolism between the mother and fetus can lead to an increase in reactive oxygen species (ROS) levels during this period, thereby causing systemic oxidative stress and inflammation [8]. Furthermore, an increase in the maternal basal metabolic rate during gestation enhances the susceptibility of both the dam and fetus to oxidative stress [9]. Specifically, sows have been shown to experience high levels of oxidative stress during the late gestation and early lactation periods, manifested by significant increases in ROS and oxidative stress markers (MDAs) [10]. Several relevant studies also indicate that low-parity sows are more susceptible to oxidative stress [11]. Notably, maternal redox imbalance during pregnancy exhibits an intricate interplay with fetal oxidative stress, a primary factor contributing to fetal abortion, premature farrowing, low birth weight, congenital abnormalities, and impaired immune function [12,13,14]. Meanwhile, the placenta serves as a bridge mediating the stress response [15,16]. However, oxidative stress can affect the normal functions of the placenta, such as blood-vessel formation, structural integrity, and the exchange of substances between the mother and fetus [17]. For instance, ROS can cause damage to the fetus’s growth and development through the placenta, leading to fetal death and intrauterine growth retardation (IUGR) and ultimately impairing reproductive capacity [18]. The intestinal epithelium, a mucosal barrier consisting of a single layer of cells, maintains the intestinal luminal environment and regulates the penetration of harmful substances (e.g., bacteria, toxins, and dietary antigens) into enterocytes [19,20]. The maintenance of intestinal homeostasis depends on intestinal epithelial integrity, which is supported by junctional proteins that connect adjacent epithelial cells and form a physical barrier [21]. Additionally, the gastrointestinal tract (GI) is a major source of ROS. Oxidative stress induced by ROS accumulation can alter the structure of tight-junction proteins, thereby increasing intestinal permeability. Oxidative stress can also induce intestinal inflammation and apoptosis of intestinal mucosal epithelial cells, thereby impairing the intestinal mucosal barrier [22]. Thus, inhibiting oxidative stress or proinflammatory cytokines can protect intestinal barrier function [23,24].

The intake of antioxidant nutrients can enhance the antioxidant capacity of animals, and it is considered a reasonable strategy for preventing oxidative stress [25]. Soy isoflavone (SI), a type of flavonoid, is natural phytoestrogens that mainly exist in soybeans and their products. It has anti-cancer, anti-osteoporotic, and antioxidant properties [26]. Due to its ability to improve animal reproductive performance, SI has been adopted in animal production [27]. SI demonstrate strong antioxidant capacity both in vivo and in vitro. In in vivo studies, dietary supplementation with daidzein (one of the major active components of soy isoflavones) has been shown to enhance the activity of key antioxidant enzymes in swine, thereby improving their overall antioxidant capacity [28,29]. In in vitro studies, SI can enhance the expression of several antioxidant genes (such as HO-1 and NQO1) by activating the Nrf2 signaling pathway, thereby improving the antioxidant capacity of porcine intestinal epithelial cells (IPEC-J2) to counteract the oxidative damage induced by ROS. Additionally, SI upregulates the expression of tight-junction proteins (ZO-1, Occludin) to strengthen the integrity of porcine intestinal epithelial cells [30]. Furthermore, studies have demonstrated that soy isoflavones can also improve placental function by upregulating the expression of several key functional genes in the placenta of sows (e.g., SNAT1 and IGF-1) [28]. However, a gap exists in the research on SI supplementation in the later stage of pregnancy for the intestinal health of sow fetus. This study focused on the gestational stages of sows to investigate the impact of SI on the gut health of piglets.

## 2. Materials and Methods

### 2.1. Diets and Feeding Management

This animal experiment operation complied with the regulations of the Animal Ethics Committee of the Animal Technology College of Henan Agricultural University and was approved by the Animal Protection and Utilization Committee of the University (approval number: HNND 20240710, Zhengzhou, China). The experiment was conducted at Pingdu Huayu Pig Technology R&D Co., Ltd., Qingdao, China. Forty sows at 85 days of gestation with 1–2 parities were randomly divided into control (Con; basic diet, *n* = 20) and experimental groups (SI; basic diet + 100 mg/kg of feed SI, *n* = 20). The dosage of SI added was referenced from previous studies [28,29]. The basal diet met the nutritional requirements of pregnant sows, as defined by the National Research Council (NRC, 2012). Table 1 presents the dietary and nutritional composition of the basal diet of the sows.

All the sows were fed in a separate positioning column (0.6 × 2.2 m) with unrestricted drinking water. The environment of the gestation house was controllable during the experiment, with gas exchange maintained via an air filtration system, while the air conditioning system kept the temperature within the house at approximately 20.6 °C. Pregnant sows were fed twice a day (08:00 and 17:00), with 1.4 kg per feeding and a total dietary intake of 2.8 kg per day. For the experimental group, to ensure precise SI dosing for each sow, 100 mg/kg of SI was added to the diet based on individual feed intake and thoroughly mixed before feeding. The experiment was initiated on day 85 of gestation and continued until the sows farrowed. After 109 days of pregnancy, the animals were transferred to a farrowing pen (0.6 × 2.2 m) for labor preparation. Prior to sow introduction, the farrowing house underwent rigorous cleaning and disinfection procedures to minimize the potential impact of pathogenic microorganisms. The farrowing crates were equipped with plastic woven floor sections, designated specifically for piglet resting. Each pen was furnished with heating lamps, along with ad libitum drinking devices for both sows and piglets, as well as detachable feed troughs dedicated to sows. Daily care and vaccination of the sows were performed in accordance with farm regulations.

### 2.2. Sample Collection

Upon farrowing, the total litter size, stillbirths, and mummified fetuses were recorded for each sow. Immediately after birth, the umbilical stump of piglets and its surrounding area were immediately wiped with 5% iodine tincture and allowed to air-dry naturally. Meanwhile, warm water was used to remove contaminants from the surface of sows’ mammary glands; the piglets’ body surfaces were dried with sterile towels to ensure they were clean and dry, thereby reducing pathogen contamination. Piglets’ weight was measured after birth, but before their first milk intake. In addition, the litter weight (the total weight of all live piglets per sow), weak piglet rate (number of weak piglets divided by the total number of live piglets), and coefficient of variation of weight (CV, calculated as (standard deviation of piglet weights within the litter/mean weight of piglets within the litter) × 100%) were calculated.

Upon farrowing, 10 healthy sows were randomly selected from each group, and 5 mL of blood was collected from the ear vein. The blood samples were spun at 3000× *g* for 10 min. The separated supernatant was aliquoted into enzyme-free centrifuge tubes and stored at −80 °C. Sample collection was completed within 2 min after the sow’s placenta was expelled. All placental tissues of the sows were collected from placentas corresponding to average-weight piglets. These tissues were immediately divided into several centrifuge tubes and placed in a −80 °C refrigerator for subsequent analysis.

Piglets were temporarily deprived of colostrum intake for postpartum. After all the piglets were born, we selected six piglets close to the average body weight from each group for slaughter after being anesthetized. These piglets were from different sows, and all were female. Blood was drawn from the anterior vena cava, and the plasma was isolated for further analysis. After the piglets were euthanized, the organs were separated and weighed to calculate their indices. Two portions of jejunal and ileal tissues were collected and placed in 4% paraformaldehyde for evaluation of intestinal morphology and immunofluorescence-based detection of protein expression. A section of the intestine tissue was washed with physiological saline, quickly placed in liquid nitrogen for cryopreservation and immediately transferred to a −80 °C refrigerator for storage. This was performed to determine the expression levels of related proteins.

### 2.3. Chemical Analysis

Oxidative stress-related indices of the plasma and tissue homogenates from the sows and piglets were assayed. Approximately 100 mg of tissue sample was weighed and homogenized in 1 mL of lysis buffer using a grinder (JXFSTPRP-64, Jingxin, Shanghai, China). After centrifugation at 12,000× *g* for 15 min at 4 °C, the supernatant was collected for protein quantification and oxidative stress analysis. Oxidative stress-related indices in blood or tissues, including T-AOC, SOD, CAT, GSH-PX, and MDA, can react with 2,2′-Azino-bis (3-ethylbenzothiazoline-6-sulfonic acid) (ABTS), Water-Soluble Tetrazolium Salt-1 (WST-1), ammonium molybdate (AM), H_2_O_2_, and thiobarbituric acid (TBA), respectively. Therefore, corresponding diagnostic kits were selected for detection. To assess the total antioxidant capacity (T-AOC, A015-2-1, ABTS method) and enzyme activities, including superoxide dismutase (SOD, A001-3-2, WST-1 method), catalase (CAT, A007-1-1, AM method), glutathione peroxidase (GSH-PX, A005-1-2, H_2_O_2_ method), and malondialdehyde (MDA, A003-1-2, TBA method), diagnostic kits from Nanjing Jiancheng Bioengineering Institute (Nanjing, China) were utilized in the analysis. The absorbance values of each sample were measured using a microplate reader (Thermo Fisher Scientific, Carlsbad, CA, USA).

### 2.4. Hematoxylin and Eosin Staining

Fresh jejunal and ileum tissue samples (6 per group) were immersed in 4% paraformaldehyde overnight for HE staining. Tissues were processed with alcohol and xylene and then embedded in paraffin. Embedded tissue was sectioned, dewaxed, rehydrated and stained with H&E. Different positions of each tissue sample were selected for slicing, and eventually, three sections were obtained. Images were captured with a Nikon E100 microscope, and villus height and recess depth were measured using ImageJ (version 1.4.3.67). Villus height and crypt depth were measured by randomly selecting three different visual fields from each section.

### 2.5. Western Blotting Analysis

Placental and intestinal tissues were ground in liquid nitrogen and lysed in RIPA buffer (Beyotime Biotechnology Co., Ltd., Shanghai, China) with 1 mM PMSF (Beyotime Biotechnology Co., Ltd., Shanghai, China) and 1% phosphatase inhibitor. Thirty micrograms of protein was separated by SDS-PAGE and transferred to PVDF (MilliporeSigma, Billerica, MA, USA). The membrane was washed with a rapid sealing solution (Beyotime Biotechnology Co., Ltd., Shanghai, China) and subsequently washed four times with TBST (Beijing Pulilai Gene Technology Co. Ltd., Beijing, China) buffer. It was incubated with the primary antibody at 4 °C for 12–15 h, then washed three times with TBST, and subsequently incubated with the secondary antibody at room temperature for 1 h. After incubation, the antibody was washed three times with TBST buffer. The ECL Plus chemiluminescence detection kit (Beijing Pulilai Gene Technology Co., Ltd., Beijing, China) and chemiluminescence imaging analysis system (Tanon Gel Imaging System, Shanghai, China) were used to capture the signals on the film. ImageJ (v1.4.3.67) was used for grayscale analysis. The expression level was standardized using β-actin as a reference. In this study, we used anti-NrF2, anti-GPX4, anti-HO-1, anti-NQO1, anti-ACSL4, anti-XCT, anti-HSP70, anti-Bax, anti-BCL2, anti-caspase 3, anti-ZO-1, anti-claudin, anti-occludin, and anti-β-actin antibodies (Wuhan Sanying Biotechnology Co. Ltd., Wuhan, China). Additionally, anti-rabbit IgG, anti-mouse IgG, and anti-β-actin antibodies were purchased from Amy Jet Scientific (Wuhan, China).

### 2.6. Tissue Immunofluorescence

The placental tissues of sows and jejunal tissues of newborn piglets were collected, fixed in 4% paraformaldehyde (24 h), dehydrated with sucrose, and embedded in OCT for sectioning at a 10 μm thickness. Then, they were permeabilized with 0.1% Triton (10 min), blocked with 5% BSA (1 h) and incubated overnight with rabbit GPX4 and Nrf2 (1:200) at 4 °C. Next day, they were washed three times with PBS and incubated with Alexa Fluor 488 anti-rabbit (1:500) at RT in the dark for 1 h. The nuclei were stained with DAPI (1:1000, 10 min), and samples were sealed with an anti-quenching agent. Fluorescence images were obtained using a Nikon fluorescence microscope.

### 2.7. Statistical Analysis

Data were analyzed with GraphPad Prism 8.0 (San Diego, CA, USA) or SPSS 26.0 (IBM, Armonk, NY, USA). The data obtained were subjected to the Shapiro–Wilk and Levene’s tests for normal distribution and homogeneity of variances, respectively. On the premise that the data met the requirements of normal distribution and homogeneity of variances, a two-tailed unpaired t-test was used to analyze differences between the two groups. The results with a probability value of <0.05 were considered statistically significant, whereas those with a probability value of 0.05 ≤ *p* < 0.10 were considered a tendency.

## 3. Results

### 3.1. Effects of Dietary SI Supplementation on the Reproductive Performance of Sows

Table 2 shows that there were no significant differences in the total litter size, the number of stillbirths, birth weight, litter weight, the rate of weak piglets, and the birth weight of newborn piglets between the groups (*p* > 0.05). However, the number of stillbirths showed a decreasing trend (*p* = 0.082) in the SI treatment group.

### 3.2. Effects of Dietary SI Supplementation on the Organ Indices of Newborn Piglets

Table 3 shows that supplementing SI to sows during the late stage of pregnancy did not cause significant changes in the weight indices of the heart, spleen, lungs, or kidneys (*p* > 0.05). However, the intestinal organ index (*p* < 0.05) and liver organ index (*p* < 0.05) of the newborn piglets in the SI group significantly increased.

### 3.3. Effects of Dietary SI Supplementation on the Intestinal Morphology of Newborn Piglets

**Figure 1:**
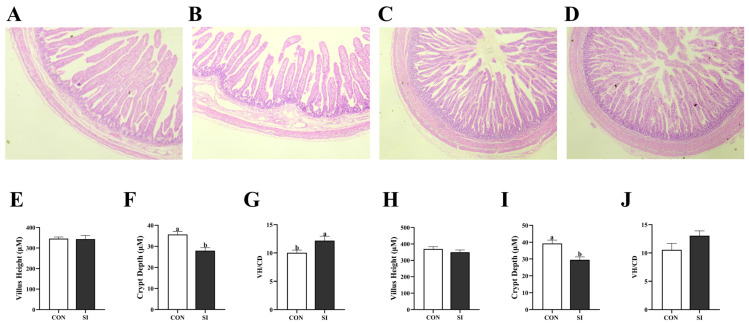
Effects of dietary SI supplementation on the morphology of the jejunum and ileum of the newborn piglets: (**A**) Con jejunum; (**B**) SI jejunum; (**C**) Con ileum; (**D**) SI ileum; (**E**–**G**) quantitative data for the jejunum; (**F**–**J**) quantitative data for the ileum. Con—control; SI—soy isoflavone; VH/CD—ratio of villus height to crypt depth. The data are presented in the form of mean ± standard error (*n* = 6). Different letters indicate statistically significant differences.

Figure 1 demonstrates that, in the jejunum, the SI group exhibited no significant difference in villus height *(p* > 0.05; Figure 1E), while crypt depth decreased (Figure 1F) and the villus height/crypt depth ratio increased (*p* < 0.05; Figure 1G). In the ileum, crypt depth in the SI group decreased (*p* < 0.05; Figure 1I), whereas neither villus height nor the villus height/crypt depth ratio differed significantly (*p* > 0.05; Figure 1H,J).

### 3.4. Effects of Dietary SI Supplementation on the Antioxidant Capacity of Sows and Piglets

As presented in Table 4, when compared to the control group, the activities of T-AOC and SOD in the serum of sows in the SI group were significantly increased (*p* < 0.05). The activities of T-AOC (*p* < 0.05) in the sow placenta significantly increased.

As presented in Table 5, the contents of T-AOC, CAT, and GSH-Px in the serum of newborn piglets in the SI group were significantly increased. The content of MDA in the intestines of the newborn piglets in the SI group was significantly reduced (*p* < 0.05). In addition, the MDA content in the serum of newborn piglets in the SI group showed a decreasing trend (*p* = 0.094), while the GSH-Px in the intestine of newborn piglets in the SI group showed an increasing trend (*p* = 0.054).

### 3.5. Effects of SI Supplementation on Antioxidant Protein in Sow Placenta

**Figure 2:**
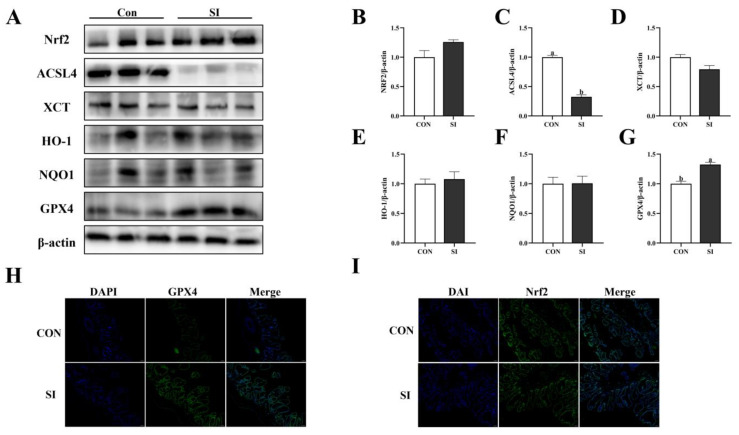
Effects of SI supplementation on antioxidant-related proteins in the placenta of sows. (**A**) Western blotting results for the Nrf2 antioxidant pathway in the placenta. (**B**–**G**) Expression of the Nrf2 antioxidant pathway in the placenta (Nrf2, ACSL4K, XCT, HO-1, NQO1, and GPX4). (**H**) Immunofluorescence analysis of GPX4 (green) and DAPI (blue) nuclear staining in the placenta. (**I**) Immunofluorescence analysis of Nrf2 (green) and DAPI (blue) nuclear staining in the placenta. Con—control; SI—soy isoflavone. Data are presented as mean ± standard error (*n* = 3). Different letters indicate statistically significant differences.

Figure 2 shows that the expression level of GPX4 protein in the SI group was significantly increased (*p* < 0.05; Figure 2G), while the expression level of ACSL4 protein in the placenta was significantly decreased (*p* < 0.05; Figure 2C). However, there were no significant differences in Nrf2 (Figure 2B), XCT (Figure 2D), HO-1 (Figure 2E), and NQO1 (*p* > 0.05; Figure 2F). The results of the tissue immunofluorescence also showed that the fluorescence intensity of GPX4 protein in the placental SI group significantly increased, while the fluorescence intensity of Nrf2 protein did not show any significant change.

### 3.6. Effects of SI Supplementation on the Apoptotic Proteins and Tight-Junction Proteins in the Placenta of Sows

**Figure 3:**
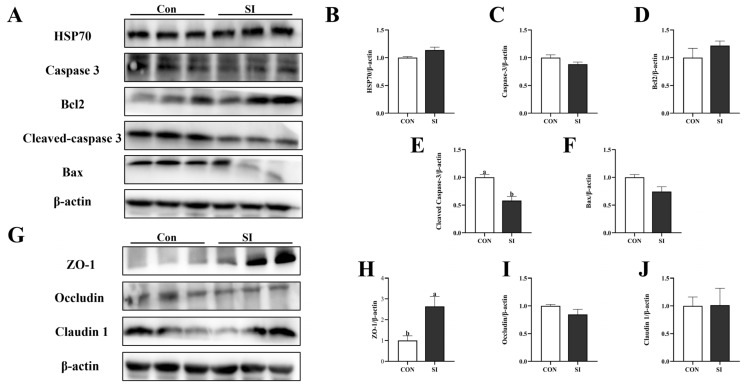
Effects of SI supplementation on placental apoptosis and tight-junction-related proteins in sows. (**A**) Western blotting results for apoptosis-related and heat-stress proteins in the placenta. (**B**–**F**) Expression of apoptosis-related and heat-stress proteins (HSP70, caspase 3, Bcl2, cleaved caspase, and Bax) in the placenta. (**G**) Western blotting results for tight-junction proteins in the placenta. (**H**–**J**) Expression of placental tight-junction proteins in the placenta (ZO-1, occludin, and claudin 1). Con—control; SI—soy isoflavone. Data are presented as mean ± standard error (*n* = 3). Different letters indicate statistically significant differences.

Figure 3 shows that adding SI to diet of sows during the late gestation significantly reduced the expression of cleaved Caspase 3 protein in the placenta (*p* < 0.05; Figure 3E) and significantly increased the expression of ZO-1 protein (*p* < 0.05; Figure 3H) but had no effect on the expression of HSP70 (Figure 3B), Caspase3 (Figure 3C), Bcl2 (Figure 3D), Bax (Figure 3F), Occludin (Figure 3I), and Claudin1 proteins (*p* > 0.05; Figure 3J).

### 3.7. Effects of SI Supplementation on the Antioxidant Proteins in the Intestinal Tract of Newborn Piglets

**Figure 4:**
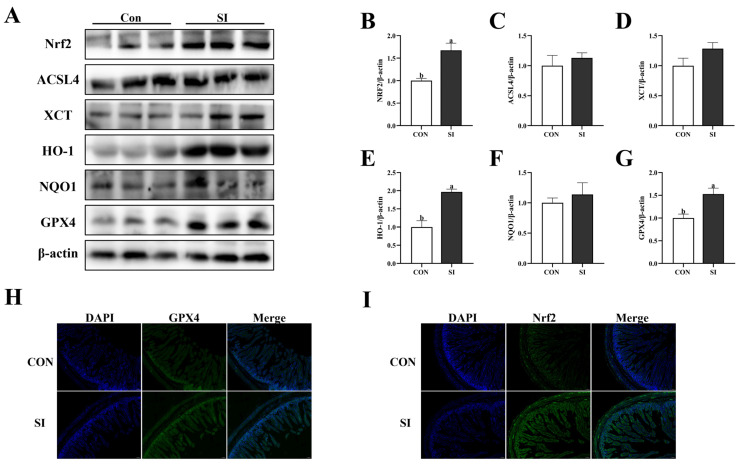
Effects of SI supplementation on antioxidant-related proteins in the jejunum of piglets. (**A**) Western blotting results for the Nrf2 antioxidant pathway in the jejunum. (**B**–**G**) Expression of the Nrf2 antioxidant pathway in the jejunum (Nrf2, ACSL4K, XCT, HO-1, NQO1, and GPX4). (**H**) Immunofluorescence analysis of GPX4 (green) and DAPI (blue) nuclear staining in the jejunum. (**I**) Immunofluorescence analysis of Nrf2 (green) and DAPI (blue) nuclear staining in the jejunum. Con—control; SI—soy isoflavone. Data are presented as mean ± standard error (*n* = 3). Different letters indicate statistically significant differences.

Figure 4 shows that the addition of SI during the late stage of pregnancy significantly increased the proteins expression of Nrf2 (Figure 4B), HO-1 (Figure 4E), and GPX4 (*p* < 0.05; Figure 4G) in the jejunum of newborn piglets. However, it had no significant effect on the protein expression levels of ACSL4 (Figure 4C), XCT (Figure 4D), and NQO1 (*p* > 0.05; Figure 4F). The results of the tissue immunofluorescence also showed that the fluorescence intensities of GPX4 (Figure 4H) and Nrf2 (Figure 4I) proteins in the piglets’ intestines of the SI group were significantly increased.

### 3.8. Effects of SI Supplementation on the Apoptosis Proteins and Tight-Junction Proteins in the Intestine of Newborn Piglets

**Figure 5:**
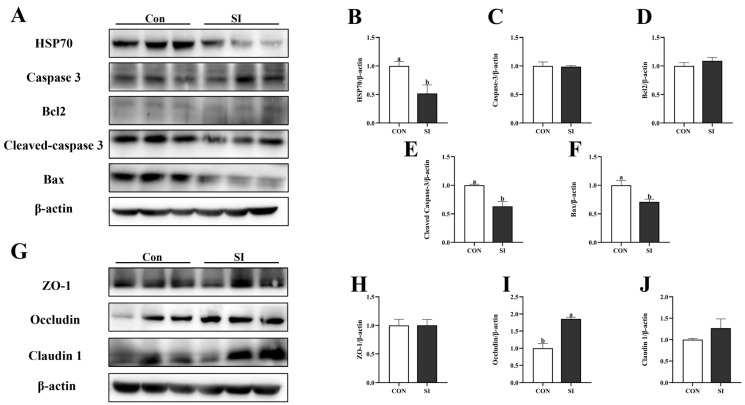
Effects of SI supplementation on apoptosis in the jejunal mucosa and the expression of tight-junction-related proteins in piglets. (**A**) Western blotting results for apoptosis-related and heat-stress proteins in the jejunum. (**B**–**F**) Expression of apoptosis-related and heat-stress proteins in the jejunum (HSP70, caspase 3, Bcl2, cleaved caspase, and Bax). (**G**) Western blotting results for tight-junction proteins in the jejunum. (**H**–**J**) Expression of jejunum tight-junction proteins in the jejunum (ZO-1, occludin, and claudin 1). Con—control; SI—soy isoflavone. Data are presented as mean ± standard error (*n* = 3). Different letters indicate statistically significant differences.

Figure 5 shows that the expression levels of HSP70 protein (Figure 5B), Bax protein (Figure 5F), and cleaved Caspase 3 protein (*p* < 0.05; Figure 5E) in the SI group of newborn piglets were significantly downregulated, while the expression level of Occludin was significantly increased (*p* < 0.05; Figure 5I). There were no significant differences in the expression levels of Caspase 3 protein (Figure 5C), Bcl2 protein (Figure 5D), ZO-1 (Figure 5H), and Claudin 1 (*p* > 0.05; Figure 5J).

## 4. Discussion

The growth requirements of the fetus and developmental requirements of the placenta cause an increase in the metabolic rate of sows during the late stage of pregnancy. This leads to the accumulation of ROS, thereby rendering the fetus more susceptible to oxidative stress [31]. The use of functional nutrients is considered a good strategy to prevent oxidative stress. Studies have shown that supplementation with low or moderate doses of SI before farrowing can significantly improve the antioxidant capacity and reproductive performance of sows [28]. MDA is formed when polyunsaturated fatty acids are oxidized by free radicals. MDA levels directly reflect the degree of oxidative damage and serve as a typical indicator of oxidative stress [32]. GSH-px, an important antioxidant enzyme in animals, protects cells from oxidative damage by catalyzing the reduction of peroxides (such as H_2_O_2_ or organic peroxide) by glutathione (GSH) [33]. Superoxide dismutase (SOD) and catalase (CAT) synergistically scavenge reactive oxygen species (ROS) to protect cells from oxidative damage [34]. T-AOC is used to assess the overall antioxidant capacity. Given that low-parity sows are more sensitive to oxidative stress [11], in this experiment, we selected low-parity sows as the research subjects. This study measured antioxidant oxidase activity (GSH-PX, CAT, and SOD) and markers of oxidative stress (MDA) in the plasma and tissues of sows and piglets. The results showed that the SI supplementation group significantly elevated the activity of antioxidant enzymes in the sows’ blood (T-AOC and SOD) and placenta (T-AOC), which is consistent with previous studies [28]. These results indicated that SI enhanced the antioxidant capacity of sows. It has been shown that maternal nutritional interventions can affect the growth performance and antioxidant capacity of the offspring [25,28]. This study further confirms this conclusion, as measurements of antioxidant capacity in piglet serum showed that supplementing sows’ diets with SI increased the activities of T-AOC, CAT, and GSH-Px.

Oxidative stress can affect the reproductive performance of sows, placental function, and intrauterine development of the fetus [16]. Consistent with these observations, our results showed a downward trend in the stillbirth rate. The liver is a key metabolic and immune organ, with its development closely linked to pig growth [35,36]. The gut is not only the center of nutrient absorption but also a key organ that resists pathogens and maintains immune balance [37]. Eighty percent of immune cells are located in the gut, and intestinal flora directly affect immune response and metabolic processes in animals, synthesizing key metabolites such as vitamin K and short-chain fatty acids (SCFAs) [38]. In this study, the intestinal and liver organ indices in the SI group were significantly elevated. These findings indicate that SI can promote the development of intestinal and hepatic systems during the fetal stage. Intestinal histomorphology reflects the health of the intestine, as well as digestion and nutrient absorption. The ability to digest and absorb intestinal nutrients primarily depends on the integrity of the villus–crypt structure of the intestine [39]. Li reported that SI can increase the ratio of intestinal villus height to crypt depth, thereby protecting intestinal health [29]. Our results are consistent with theirs: supplementation with SI during late pregnancy improves the intestinal morphology and structure of fetuses.

Nrf2, a redox-sensitive transcription factor, orchestrates the cellular antioxidant defense and detoxification systems by transactivating genes harboring antioxidant response elements (AREs). Concurrently, it modulates the expression of ferroptosis-related downstream genes such as GPX4 and ACSL4. Activation of Nrf2 plays a pivotal role in mitigating ferroptosis [40,41]. Glutathione is an antioxidant used to maintain the balance between oxidation and reduction in cells [42]. GPX4 utilizes glutathione as a cofactor to reduce lipid peroxides to lipid alcohols, thereby inhibiting lipid peroxidation [43]. ACSL4 plays a key role in ferroptosis and reduced its expression alleviates lipid peroxide accumulation by inhibiting ferroptosis [44]. Xu (2023) demonstrated that methionine inhibits heat stress-induced ferroptosis in bovine mammary epithelial cells by activating the Nrf2 signaling pathway to stimulate GPX4 expression [45]. Our results showed that the protein expression levels of GPX4 in the placenta and intestinal tract of the newborn piglets were significantly increased, that of placental ACSL4 was significantly decreased, and those of Nrf2 and HO-1 in newborn piglets were significantly increased. These results suggested that SI played an important role in preventing ferroptosis in the placenta and intestines.

The placenta acts as the core junction between the sow and the fetus [46]. It mediates the absorption and uptake of nutrients such as glucose and amino acids in the material cycle of the sow and fetus, coordinates the exchange of oxygen between the sow and fetus and eliminates fetal waste. These functions are essential for fetal development in sows; therefore, optimal placental function is critical for healthy fetal development [47]. Wu (2023) showed that SI exhibits antioxidant and anti-apoptotic properties and inhibits doxorubicin (Dox)-induced cardiac injury in rats [48]. Additionally, SI exhibits a protective effect against lipopolysaccharide (LPS)-induced intestinal epithelial barrier damage [42]. These studies suggested that SI has antiapoptotic effects, consistent with our results. In our study, apoptotic protein levels in the fetal placenta and jejunum of the SI group were reduced. Timely downregulation of HSP70 can prevent the cytotoxicity caused by its excessive accumulation [49]. In our study, HSP70 protein levels in the intestines of piglets were significantly downregulated, possibly related to the fact that SI protects the intestines of piglets and alleviates the heat stress in the womb. As crucial proteins in tight junctions, ZO-1, Occludin, and claudin 1 are vital to intestinal and placental [50,51,52]. The barrier integrity of the placenta and intestine can effectively prevent bacterial invasion. Li (2022) showed that SI can improve the barrier function of intestinal cells [30]. The protein expression levels of Occludin in the intestine and ZO-1 in the placenta were significantly increased. These results indicate an improvement in the function of the placental barrier in the sows and intestinal barrier in the newborn piglets. This suggests that SI enhanced the health of the placenta of the sow and piglet intestines.

## 5. Conclusions

The supplementation of SI in sows’ diets during late gestation did not affect the number of newborn piglets, birth weight, or the rate of weak piglets. However, it enhanced the antioxidant status of sows and newborn piglets by regulating antioxidant enzyme activity while improving piglets’ intestinal health. These effects may be linked to SI activating the Nrf2 signaling pathway in the placenta and piglet intestines, thereby promoting the expression of downstream antioxidant proteins.

## Figures and Tables

**Table 1:** Ingredients and nutrient content of diets for sows in late gestation (as fed basis).

Ingredients	Proportion, %
Corn	59.14
Soybean meal	21.00
Wheat bran	15.00
Soybean oil	1.00
Limestone	1.10
Dicalcium phosphate	1.20
Salt	0.40
Choline chloride	0.15
L-Lysine·H_2_SO_4_ (70%)	0.32
DL-Methionine (99%)	0.03
L-Threonine (99%)	0.16
Vitamin and mineral premix ^a^	0.50
Chemical composition ^b^	
Crude protein, %	16.94
Digestible energy, Mcal/kg	3.20
Net energy, Mcal/kg	2.31
Calcium, %	0.77
Total phosphorus, %	0.64
Available phosphorus, %	0.35
Standardized total tract digestible phosphorus	0.37
Standardized ileal digestible Lysine, %	0.87
Standardized ileal digestible Methionine, %	0.26
Standardized ileal digestible Threonine, %	0.68
Standardized ileal digestible Tryptophan, %	0.18
Standardized ileal digestible Valine, %	0.69

^a^ Provided per kg of diet: vitamin A, 9000 IU; vitamin D3, 1000 IU; vitamin E, 86.2 IU; vitamin K3, 1.5 mg; vitamin B1, 1.0 mg; vitamin B2, 4.75 mg; vitamin B6, 3.5 mg; vitamin B12, 20 μg; niacin, 20 mg; pantothenic acid, 20 mg; folic acid, 0.85; copper, 15 mg; iron, 100 mg; zinc, 110 mg; manganese, 30 mg; selenium, 0.2 mg; iodine, 0.3 mg. ^b^ Calculated values.

**Table 2:** Effects of dietary SI supplementation on the reproductive performance of sows.

Item	Con	SI	SEM	*p*-Value
Total litter size	15.83 ± 0.67	14.88 ± 0.84	0.53	0.379
Stillbirth	1.28 ± 0.33	0.64 ± 0.16	0.18	0.082
Birth weight (kg)	1.34 ± 0.02	1.33 ± 0.03	0.02	0.661
Litter weight (kg)	18.93 ± 0.77	18.26 ± 0.78	0.55	0.557
Weak piglet rate (%)	0.12 ± 0.02	0.11 ± 0.02	0.01	0.733
CV weight%	0.20 ± 0.01	0.19 ± 0.02	0.01	0.564

Con—control; SI—soy isoflavone; the data are presented in the form of mean ± standard error (*n* = 20); weak piglet rate—piglets with a body weight below 1 kg; CV _weight_—within-litter body weight variation in neonatal piglets.

**Table 3:** Effects of dietary SI supplementation on the organ indices of newborn piglets (g/kg).

Item	Con	SI	SEM	*p*-Value
Heart	6.52 ± 0.30	6.66 ± 0.36	0.47	0.626
Liver	23.27 ± 1.09	28.90 ± 1.31	1.71	0.008
Lung	15.07 ± 0.75	14.62 ± 0.45	0.87	0.615
Kidney	7.66 ± 0.40	7.65 ± 0.41	0.57	0.990
Intestine	37.75 ± 0.96	40.74 ± 0.83	1.27	0.040
Stomach	4.98 ± 0.31	5.23 ± 0.37	0.48	0.605
Spleen	0.92 ± 0.07	0.93 ± 0.07	0.10	0.919

Con—control; SI—soy isoflavone; organ indices (g/kg)—organ weight (g)/body weight (kg); the data are presented in the form of mean ± standard error (*n* = 6).

**Table 4:** Effects of dietary SI supplementation on the antioxidant indices of sows.

Item	Con	SI	SEM	*p*-Value
Serum				
T-AOC, nmol/mL	0.05 ± 0.01	0.07 ± 0.02	0.01	0.017
SOD, U/mL	41.20 ± 3.25	70.67 ± 8.18	8.80	0.008
CAT, U/mL	7.16 ± 1.47	8.73 ± 1.19	1.47	0.314
GSH-Px, U/mL	723.64 ± 49.63	775.97 ± 42.42	69.29	0.436
MDA, nmol/mL	3.54 ± 0.26	2.86 ± 0.35	0.44	0.144
Placenta tissue				
T-AOC, nmol/mg prot	0.84 ± 0.09	1.32 ± 0.18	0.21	0.038
SOD, U/mg prot	4.86 ± 1.20	3.98 ± 0.55	1.31	0.517
CAT, U/mg prot	56.38 ± 5.32	57.46 ± 8.02	9.63	0.912
GSH-Px, U/mg prot	88.89 ± 23.13	108.48 ± 7.51	24.32	0.432
MDA, nmol/mg prot	2.88 ± 0.42	3.53 ± 0.48	0.64	0.321

Con—control; SI—soy isoflavone; T-AOC—total antioxidant capacity; SOD—superoxide dismutase; CAT—catalase; GSH-PX—glutathione peroxidase; MDA—malondialdehyde; the data are presented in the form of mean ± standard error (*n* = 8).

**Table 5:** Effects of dietary SI supplementation on the antioxidant indices of newborn piglets.

Item	Con	SI	SEM	*p*-Value
Serum				
T-AOC, nmol/mL	0.10 ± 0.01	0.13 ± 0.01	0.01	0.014
SOD, U/mL	66.84 ± 4.30	65.94 ± 5.24	6.78	0.895
CAT, U/mL	3.07 ± 0.53	5.77 ± 0.83	0.96	0.020
GSH-Px, U/mL	232.10 ± 18.32	344.27 ± 38.82	42.93	0.029
MDA, nmol/mL	15.40 ± 3.92	7.53 ± 1.65	4.25	0.094
Intestine tissue				
T-AOC, nmol/mg prot	0.99 ± 0.12	1.20 ± 0.07	0.14	0.180
SOD, U/mg prot	12.31 ± 1.01	10.95 ± 0.73	1.25	0.310
CAT, U/mg prot	211.58 ± 10.85	221.28 ± 10.38	42.42	0.530
GSH-Px, U/mg prot	41.62 ± 3.73	59.78 ± 7.72	8.59	0.054
MDA, nmol/mg prot	3.29 ± 0.19	2.11 ± 0.41	0.48	0.029

Con—control; SI—soy isoflavone; T-AOC—total antioxidant capacity; SOD—superoxide dismutase; CAT—catalase; GSH-PX—glutathione peroxidase; MDA—malondialdehyde; the data are presented in the form of mean ± standard error (serum: *n* = 8; intestine: *n* = 6).

## Data Availability

Original results featured in this research are contained within the article, with further questions able to be referred to the corresponding author.

## Abbreviation

MDA	Malondialdehyde
Nrf2	Nuclear Factor Erythroid 2-Related Factor 2
ACSL4	Acyl-CoA Synthetase Long-Chain Family Member 4
XCT	Cystine/Glutamate Antiporter
HO-1	Heme Oxygenase-1
NQO1	NAD(P)H: Quinone Oxidoreductase 1
GPX	Glutathione Peroxidase 4
β-actin	Beta-actin
DAPI	4′,6-Diamidino-2-Phenylindole
HSP70	Heat Shock Protein 70
Caspase 3	Cysteine-Aspartic Acid Protease 3
Bcl2	B-Cell Lymphoma 2
Cleaved-caspase 3	Cleaved Cysteine-Aspartic Acid Protease 3
Bax	Bcl-2-Associated X Protein
ZO-1	Zonula Occludens-1
